# Lung shunt fraction quantification methods in radioembolization: What you need to know

**DOI:** 10.1259/bjr.20220470

**Published:** 2022-07-25

**Authors:** Pooya Torkian, Ranjan Ragulojan, Gregory J. Woodhead, Donna D'Souza, Siobhan Flanagan, Jafar Golzarian, Shamar Young

**Affiliations:** 1 Department of Radiology, Vascular and Interventional Radiology, University of Minnesota, Minneapolis, United States; 2 Department of Medical Imaging, University of Arizona, 1501 North Campbell Avenue, Tucson, United States

## Abstract

In some patients undergoing radioembolization, lung toxicity is a limiting factor when calculating their dose. At the same time, it is known that the lung shunt fraction (LSF) is overestimated by the mapping exam. Furthermore, there are multiple methods to measure LSF. Planar measurement is both the most commonly utilized and easiest to perform, however new dosimetry software provides the ability to use more advanced 3D techniques. This paper reviews the different LSF calculation methods and elucidates the available data comparing the techniques, clinical relevance, and dose calculation.

## Introduction

Yttrium-90 (^90^Y)-based Transarterial radioembolization (TARE) has been established to be an effective treatment of primary and secondary liver cancers.^1,2^ TARE requires two procedures per treatment. The first session is a mapping angiography to characterize the tumor and its blood vessels, followed by intraarterial delivery of 99m-Technetium macroaggregated albumin (99mTc-MAA).^1–3^ This radiotracer serves as a ^90^Y microsphere surrogate and is used to predict particle deposition within the perfused volume and calculate the lung shunt fraction (LSF). This information is utilized to confirm candidacy for TARE and calculate treatment parameters, such as the desired tumor dose. The importance of dose distributions has been emphasized over the past several years in several landmark papers.^4^ While dosimetry has rightly become an area of focus of late, accurately predicting the lung shunt fraction (LSF) calculation has not necessarily been emphasized. 99mTc-MAA is not a perfect surrogate, as there are differences in particle shape and density. Furthermore, both 99mTc-MAA and ^90^Y microsphere distribution are influenced by catheter location during delivery, vessel vasospasm, and tumor vascularity.^5,6^ These factors can ultimately lead to over or underestimation of the LSF and discrepancies between the expected and delivered lung dose (LD) by the mapping exam.

Pre-procedural LSF and lung dosimetry determination can have significant treatment implications as the threat of lung toxicity is at times a limiting factor for ^90^Y dosing, which in turn can affect outcomes. Moreover, inaccurate LSF estimation can result in procedure cancellations in patients who would otherwise benefit from ^90^Y. There are varying methods to determine LSF and lung dosimetry. The present narrative review serves to outline these methods, reflect on key studies which aim to compare them and explore the clinical significance of the differences.

## Pathophysiology of Lung Shunt Fraction

Recent advances in dosimetry and promising study outcomes have paved the way for the safe use of higher prescribed tumor doses, thus expanding the role of TARE from palliative and neoadjuvant to curative therapy. The lungs are considered a critical organ of risk during TARE treatment planning. Therefore, the efficacy of radioembolization may be compromised in patients for whom the LSF and estimated mean lung dose (MLD) derived via currently available methods inaccurately limits the desired administered activity to the liver. The aim is to deliver a tumoricidal dose to the tumor while *preserving safe limits* of *radiation* to *normal liver parenchyma* and the *lungs*.

Lung shunt is the representation of microparticles movement to the normal lung parenchyma secondary to hepatopulmonary shunt within the tumor vasculature. The microspheres are transferred from the arterial to the venous circulation through these shunts, become trapped in the lung alveoli, resulting in radiation deposition within lung tissue. High levels of radiation dose to the lung parenchyma can then induce radiation pneumonitis, a life-threatening and irreversible complication. The most commonly utilized MLD limits are 30 Gy for a single treatment and 50 Gy as a lifetime cumulative dose.^7^

## Lung dosimetry

There are two major methods to determine the LSF and in turn lung dosimetry. In both, the patient proceeds to nuclear medicine after the mapping procedure and 99mTc-MAA delivery. While in nuclear medicine, they have 2D planar scintigraphy and/or 3D single-photon emission computed tomography (SPECT) to visualize particle deposition within the liver, extrahepatic abdominal deposition and recording of lung activity. Most patients will have a SPECT/CT to determine intrahepatic distribution as well as assess the presence of extrahepatic intraabdominal deposition, but not necessarily to calculate LSF. Planar scintigraphy is both the most commonly utilized and easiest method of calculating an LSF. However, advanced dosimetry software and capable scanners provide the ability to utilize 3D SPECT/CT images and there associated higher resolution to calculate LSF. Once the nuclear images of choice are obtained, the tracer counts in both liver and lung are determined by drawing regions of interest (ROI). The LSF is then calculated using the following formula:

LSF = (Total lung count) / (Total lung count+Total liver count)

The determined LSF value can then be multiplied with the planned quantity of ^90^Y activity(A) in GBq and a conversion factor that 50 Gy of dose is generated in 1 kg of lung tissue per 1 GBq, to determine expected MLD (where total lung mass(LM_TOT)_ is arbitrarily assumed to be 1 kg)^8^ :

Mean lung dose

(MLD) = (LSF x A(GBq) x 50 (Gy*kg/ GBq))/ LM_TOT_ (kg)

Planar calculation results in two major inaccuracies. First, the determined tracer geometric count hinges on the established margins for the ROI for each organ, a task prone to difficulty and interoperator variability considering the lack of definite landmarks on planar imaging.^9,10^ Additionally, there are differing Information for Use (IFU) recommendations between the resin Sir-Spheres and glass Theraspheres generally used for ^90^Y radiotherapy. The former references utilizing the mean count values of ROIs drawn in anterior and posterior images of both lung and liver. The latter only advocates for delineating the lung ROI and utilizing the total tracer count in the field-of-view as a surrogate for the sum of lung and liver tracer count.^9^ This issue of ROI variability is compounded by the poor spatial resolution of γ scintigraphy, which is affected by scatter and respiratory motion leading to misregistration of liver tracer count within the lung ROI at the right lung base. The general outcome of these issues is overestimation of LSF.^9^ Planar techniques have also been shown to be affected by location of the liver lesion, for instance lesions located in the dome of the liver result in greater LSF overestimation.^9,11^ Secondly, the arbitrary assumed 1 kg weight for lung tissue is likely excessive as recent studies have demonstrated that CT-based calculation of lung masses among patients is on average 800 g.^12^ A prior study demonstrated that on average the extent of overestimated LSF outweighs the extent of overestimated lung mass and thus results in a culmination of overestimated MLD.^12^

The alternative SPECT/CT method theoretically addresses the above outlined inaccuracies of planar determination. The SPECT/CT method utilizes the same formula for LSF determination as the planar method however with the benefit that count values are based on 3D data/images as opposed to 2D. Utilization of CT correlation with SPECT tracer distribution allows for improved ease of anatomical segmentation of ROIs between liver and lungs as well as improved scatter and attenuation correction resulting in generally reduced misattribution of liver tracer count to the lungs and more accurate count magnitudes. The available CT images also allow for patient-specific determination of lung mass toward calculating the absorbed LD. However, SPECT/CT is not without potential inaccuracies. One stems from the discrepancy between instantaneous acquisition of CT images and prolonged SPECT acquisition which can result in misregistration of tracer count between organs due to respiration and motion. Similar to planar imaging, SPECT/CT is also prone to liver tracer signal at the dome leaking into lung base signal however to a lesser degree.

The primary reason for limited utilization of SPECT/CT to calculate LSF in most centers is that processing of the 3D data is more complex, time-consuming and software platforms are not widely available to calculate LSF from SPECT/CT. Also, since the initial safety data establishing lung limits were based on planar LSF calculation, there is not consensus on if a 30 Gy limit is valid for SPECT/CT calculation of LSF.^13^

## A review of available evidence

There has been a longstanding discussion on the topic as planar imaging clearly overestimates LSF. This is particularly problematic for cases which require dose reduction or cancelation due to high lung doses. The numbers depend on exact ROI demarcations which is operator-dependent so ROIs are prone to a higher level of scrutinization in these cases were the clinical impact is obvious. Overall, several studies, albeit with the use of phantom or retrospectively reviewed data, have demonstrated that LSF is overestimated to a greater degree in planar determination compared to SPECT/CT.^5,10,14–16^

A retrospective study from Elsayed et al evaluated LSF using planar and SPECT/CT in 293 consecutive patients. Although this study did not assess the number of patients who would have been eligible for standard dose TARE if SPECT/CT LSF calculations were used, results showed that mean planar LSF (8.27%) was significantly greater than mean SPECT/CT LSF (3.27%).^16^ Similar results were found in a prospective study by Dittman et al where 50 patients underwent planar and PET/CT measurements for LSF estimation prior to TARE.^10^ Median LSF obtained using planar imaging was 6.8% (range 3.4–32.3%), whereas the mean using SPECT/CT was significantly lower (median 1.9%, range 0.8–15.7; ρ < 0.0001), resulting in planar imaging estimations of LSF to be 3.6 times higher than SEPCT/CT estimations. These above studies are limited, by the lack of comparison of planar LSF and SPECT/CT 99mTc-MAA LSF to the realized LSF as measured by post ^90^Y delivery imaging.

There are many predictive factors for the magnitude of discrepancy between planar LSF to SPECT/CT LSF. Elsayad et al found that the absolute discrepancy was greater in patients with tumor size≥5 cm, those with a worse Child-Pugh score (B/C), and a planar LSF≥20%.^16^ Additionally, a retrospective study by Struycken et al found SPECT LSF determination to result in significantly reduced mean LSF compared to planar when evaluating 36 patients with planar LSF greater than 15% (25.1%±11.6 vs 16.0±9.3% (ρ < 0.001)).^15^ Higher values were also obtained for MLD and mean perfused liver dose using planar LSF when compared with SPECT/CT LSF. BMI ≥ 26, tumor size of <9 cm, and left hepatic arterial injection were identified as factors resulting in a greater discrepancy between planar LSF and SPECT/CT LSF.

When comparing LSF using planar and SPECT/CT imaging to the gold standard of post-delivery ^90^Y LSF, several phantom and retrospective studies have demonstrated that LSF using SPECT/CT imaging is more accurate than planar determination. In a phantom study designed by Kunnen et al., investigators used ^90^Y chloride to achieve an LSF of 15% and calculated LSF using PET/CT, SPECT/CT and planar imaging.^17^ Planar scintigraphy overestimated LSF by up to 23%. PET was found to be accurate only when the total activity was >200 MBq and widely overestimated LSF (up to 25%) with lower activities of ^90^Y. Bremsstrahlung SPECT overestimated LSF by up to 13% at low as well as high activities; SPECT using Monte Carlo (MC)-based reconstruction method accurately estimated LSF up to 1.3% even at low ^90^Y activities.^17^ These results are similar to another phantom study by Allred et al. In this phantom study, a ^99m^Tc-filled liver/lung phantom utilized to obtain three different shunt values was evaluated using planar and SPECT/CT imaging. SPECT/CT resulted in a more accurate LSF estimation within 13% of true value, whereas planar scintigraphy resulted in up to 44% overestimation.^5^

Two retrospective patient studies have also investigated the accuracy of planar LSF and SPECT/CT LSF to the realized post ^90^Y delivery LSF. In addition to the phantom study, Allred et al compared planar and SPECT/CT LSF values among 40 patients, demonstrating significant overestimation with planar imaging.^5^ Delay in scanning did not result in significant change in LSF values, but the likelihood of extra hepatic uptake increased in patients with a longer delay. In a subset of 28 patients, LSF values were compared using ^90^Y PET/CT-based measurements. The ^90^Y PET/CT LSF values (mean 1%, range 0.3–2.8) were similar to SPECT/CT (mean 1%, range 0.4–1.6; ρ = 0.968) measurements with AC (Attenuation Correction) and SC (Scatter Correction), but were significantly lower compared to those obtained by planar imaging (mean 4.1%, range 1.2–15.0, ρ = 0.0002).^5^

In addition to LSF, planar imaging also has been found to overestimate MLD and lung mass. Lopez et al compared planar LSF (which presumes lung mass of 1 kg) with LSF measured using SPECT/CT and patient specific lung mass calculated via chest CT in 52 consecutive patients.^12^ The authors found the calculated lung mass, LSF and MLD were significantly lower when compared to measurements using planar imaging, with relative mean (±SD) differences of 20% (±16%) for lung mass, 63% (±15%) for LSF and 53% (±23%) for MLD. The estimated 1-sigma uncertainties (measurement errors) for lung mass, LSF and LMD were 9%, 10%, and 13%, respectively.^12^ This new model of using SPECT/CT imaging proposed by Lopez et al for calculating LSF and MLD holds clinical significance in treatment planning for ^90^Y radioembolization procedures.

Other studies have also shown that planar LSF overestimation has clinical impact by resulting in unnecessary dose reductions and cancelation of ^90^Y radioembolization due to high lung doses. In the 36 patients retrospectively evaluated by Struycken et al, 14 had >20% planar LSF. Among these 14 patients, five patients had <20% SPECT/CT LSF and would have been eligible for upfront TARE. Similarly, seven (7/29, 24.1%) patients underwent dose reductions based on planar LSF; six of these could have received standard radioembolization dose if SPECT/CT LSF were utilized to guide management.^15^ The study is limited, although, by the fact that comparison to the gold standard, post-delivery ^90^Y LSF and the definition of cancelation at>20% LSF or reduction at >10% LSF, is not consistent with modern practice. Similarly, in the prospective study by Dittman et al, 10 patients (10/50, 20%) had planar LSF estimates of ≥10% with 2 of the 10 (20%) patients showing planar LSF >20%, however, only the 2 patients with planar LSF >20% were found to have SPECT/CT LSF >10%. Dose reduction or contraindication to TARE would have been required in 20% patients (10/50) on the basis of planar imaging, but only in 4% (2/50) of patients if SPECT/CT LSF was considered.^10^ The use of 20 or 10% instead of 30 Gy per treatment again limits this study, however.

It is important to note that timing of administration of the 99mTc-MAA relative to the imaging acquisition can impact LSF determination in both planar and SPECT/CT methods. It has been demonstrated that prolonged duration between the latter and former may lead to marked degradation of tracer universally and with an overall effect of overestimation of LSF.^18^ An additional disadvantage common to MLD determination stems from the difference in size range between the 99mTc-MAA particles and the radioactive ^90^Y microspheres, as the 99mTc-MAA are smaller, thus resulting in a potentially altered biodistribution.^19^This discrepancy was studied by Elschot et al who demonstrated lower and more accurate MLD values obtained during planning with institutionally developed Ho microspheres, more closely resembling ^90^Y microsphere size, relative to MAA MLD determination when evaluated against post-treatment Ho-microsphere dose.^19^ Of course, pre-planning treatment MLD determination with ^90^Y particle would be ideal considering it is the particle widely used for radiotherapy delivery. However currently ^90^Y bremsstrahlung SPECT and PET at doses considered safe for planning are of poor image quality and low positron emission activity, respectively, contributing to potential inaccuracies of the calculated LD with these methods.^19^

## Clinical significance

Evidence demonstrates that tumor necrosis following ^90^Y TARE is contingent on sufficient tumor dose delivery^20,21^; Vouche et al first demonstrated a significantly increased rate of complete pathological necrosis at tumor doses exceeding 190 Gy^20,22^ potentially improving tumor control, survival benefits and successful bridging to transplant. With the advent of improved liver dosimetry methods which allow for the confident delivery of greater treatment dosages, the impact of inaccurate/overestimated LSF becomes more relevant as it may be the limiting factor in overall dosage ultimately delivered.^23,24^ The current recommendations warn against exceeding LSF of 20%, single MLD of 30 Gy and cumulative MLD of 50 Gy for the prevention of radiation pneumonitis.^25^ Although this recommendation incorrectly assumes uniform dose distribution to the lung and is brought into question by evidence in the literature, such as a study of 58 patients who exceeded an MLD of 30 Gy and did not develop radiation pneumonitis,^26,27^ clinicians are undoubtedly influenced by the proposed current limit. Thus, an overestimated LSF can negatively influence dose delivery to a patient per session and limit the number of overall sessions, with potentially far-reaching consequences in terms of survival. Thus due to the relatively improved accuracy of SPECT/CT over Planar determination of LSF, it becomes advisable to utilize the former method in high LSF cases to optimize radiotherapy delivery. Future prospective studies are needed to investigate the effect of LSF determination method on radiation dose delivery and survival outcomes.
